# Baicalin mitigates alcoholic-associated liver disease via SOCS1-driven reprogramming of macrophages

**DOI:** 10.1186/s13020-025-01110-4

**Published:** 2025-05-13

**Authors:** Sha Huang, Yuhua Wang, Jinjie Wen, Wenjuan Ji, Qiuxiang Zeng, Kaili Deng, Min Li, Shanshan Kuang, Wen Zhang, Mo Chan, Chuying Zhou, Zhiping Lv, Shaohui Huang

**Affiliations:** 1 School of Traditional Chinese Medicine, Southern Medical University, Guangzhou, 510515 Guangdong China; 2 Southern Medical University Hospital of Integrated Traditional Chinese and Western Medicine, Southern Medical University, Guangzhou, 510315 China; 3 Guangdong Provincial Key Laboratory of Chinese Medicine Pharmaceutics, School of Traditional Chinese Medicine, Southern Medical University, Guangzhou, 510515 China; 4 Guangdong Basic Research Center of Excellence for Integrated Traditional and Western Medicine for Qingzhi Diseases, Guangzhou, 510515 China; 5 Department of First Clinical Medical College, Guangdong Pharmaceutical University, Guangzhou, 510006 Guangdong China

**Keywords:** ALD, Baicalin, SOCS1, Macrophage, Reprogramming

## Abstract

**Backgrounds:**

Alcoholic liver disease (ALD), a consequence of excessive alcohol consumption, is characterized by high incidence and mortality rates. Presently, there are no effective pharmacological interventions available for the treatment of ALD. Baicalin (BA), a natural flavonoid derived from the root of Scutellaria baicalensis, has exhibited notable hepatoprotective effects. Nevertheless, the mechanisms through which BA influences the interaction between suppressor of cytokine signaling 1 (SOCS1) and macrophages during hepatic immune development remain insufficiently understood.

**Materials and methods:**

This study seeks to examine the regulatory effects of BA on ALD and to elucidate the relationship between SOCS1 and macrophage differentiation. Our experimental methodology involves the novel application of zebrafish as an in vivo model for ALD. To further investigate the underlying mechanisms, we employed gene knockout and overexpression techniques.

**Results:**

The study demonstrates that BA substantially alleviates ALD in both in vivo and in vitro settings by upregulating SOCS1 expression in macrophages. Furthermore, we elucidated the association between SOCS1 and macrophage reprogramming. Specifically, SOCS1 knockdown led to the downregulation of CD86, CD80, and iNOS expression, whereas SOCS1 overexpression enhanced the expression of CD206, CD163, IL-4, and IL-10.

**Conclusion:**

In conclusion, our findings suggest that BA attenuates ALD via the modulation of SOCS1-mediated macrophage reprogramming.

**Supplementary Information:**

The online version contains supplementary material available at 10.1186/s13020-025-01110-4.

## Highlights

- BA effectively alleviates lipid accumulation and liver damage in zebrafish with ALD.
- BA significantly ameliorates ALD in vivo and in vitro by increasing the SOCS1 expression on macrophages.
- SOCS1 promotes the transformation of M1 macrophages into M2 macrophages.

**Supplementary Information:**

The online version contains supplementary material available at 10.1186/s13020-025-01110-4.

## Introduction

Alcoholic liver disease (ALD), stemming from extended periods of heavy drinking, is a major health concern around the world. Regarding its significant frequency and associated mortality, ALD has become a global health risk [1]. Data from the World Health Organization (WHO) indicates that excessive alcohol use is responsible for approximately 3.3 million deaths annually, making up 6% of global mortality [2]. ALD progresses through multiple intricate stages, beginning with steatosis and advancing to alcoholic hepatitis, liver fibrosis, cirrhosis, and potentially hepatocellular carcinoma [3]. Presently, typical treatments for ALD involve refraining from alcohol and using medication. Treatment for primary alcoholic steatosis and mild alcoholic hepatitis involves abstaining from alcohol and using medication [4]. Liver transplantation is necessary for severe cases, highlighting the urgent need to find new ALD treatments with minimal side effects.

Inflammation plays a crucial role in the advancement from initial to severe stages of ALD and has been a focus of therapeutic research for many years, yet no successful inflammatory targets have been discovered yet [5, 6]. Macrophages located in the liver are essential for the inflammatory response and ALD, as they produce different cytokines [7]. Nonetheless, the heterogeneity of liver macrophages leads them to have varying roles in the development of ALD. Recent studies have shown that changes in macrophage polarization play a crucial role in various phases of ALD. Focusing on particular macrophage subsets could offer novel strategies for treating liver failure and fibrosis in ALD. The suppressor of cytokine signaling (SOCS) family proteins are a group of regulatory proteins that provide negative feedback in various cytokine signaling pathways [8, 9]. Research indicates that in cancerous conditions, SOCS family proteins can create a negative feedback loop to suppress the JAK/STAT signaling pathway and influence macrophage polarization [10]. In addition, recent researches have also shown that conditional knockout of the *Socs1* gene in hepatic stellate cells promotes the numbers of Ly6 C^hi^CCR2^+^ pro-inflammatory macrophages in liver fibrosis [11]. However, the relationship between SOCS1 and macrophages in ALD has not been clarified, and further research is needed.

Traditional Chinese herbs have recently emerged as an essential source for drug discovery and application, attributed to their unique molecular structures and potential biotherapeutic properties. Baicalin (7-glucuronic acid, 5, 6-dihydroxyflavone, BA) serves as a prominent flavonoid ingredient in Chinese medicine, sourced from the herb *Scutellaria Baicalensis*. Numerous in vitro and in vivo studies have shown that BA has the hepatoprotective effects [12]. Researches have shown that BA treatment effectively suppressed liver inflammation induced by a methionine and choline-deficient (MCD) diet in a nonalcoholic fatty liver disease (NAFLD) mouse model [13, 14]. BA significantly reduces cholesterol and free fatty acid levels and also decreases liver lipid buildup in rats fed a high-fat diet [15]. These findings suggest that BA is increasingly seen as a potential natural remedy for ALD, and further research is needed to understand its therapeutic effects.

This research utilized zebrafish models in vivo and cell models in vitro to explore the possible molecular mechanisms and application potential of BA in ALD. We find that BA can inhibit lipid accumulation in zebrafish and liver cells, and elucidate the mechanism by which BA regulates macrophage polarization through SOCS1 to reduce alcoholic liver injury.

## Materials and methods

### Reagents and antibodies

BA (#B20570, HPLC ≥ 98%) was purchased from Shanghai yuan ye Bio-Technology. Primary antibodies against FASN(#10624-2-AP), CD80 (#66406-1), CD206 (#60143-1-lg), IL-6 (#21865-1-AP), GAPDH (#60004-1-Ig), β-actin (#66009-1-Ig), iNOS (#22226-1-AP), CD163 (#16646-1-AP), α-SMA (#67735–1-Ig), F4/80 (#29414-1-AP) were bought from Proteintech Technology. CD86 (#501199) were bought from ZENBio Technology. IL-4 (#bs-0581R), IL-2 (#bs-1191R) were bought from Bioss Technology. SOCS1 (#sc-518028) were bought from Santa Technology.

### Experimental animals and ethics statement

The zebrafish raising methods used in this study are the same as in previous research. At 4 days post-fertilization (dpf), the larvae were randomly assigned to five different groups:

1. Normal control group.
2. 350 mM ethanol group (Model).
3. 350 mM ethanol + 6.25 μM BA group.
4. 350 mM ethanol + 12.5 μM BA group.
5. 350 mM ethanol + 25 μM BA group.

Embryos in the model group were cultured in water containing 350 mM ethanol for 32 h. The drug-treated group received 6.25 μM, 12.5 μM, and 25 μM BA for 48 h. Daily monitoring was conducted on the survival rate of the drug-treated group and the water level in the dosing system.

All procedures received approval from the Southern Medical University Institutional Animal Care and Ethics Committee.

### Histological staining of zebrafish

For H&E, zebrafish larvae were fixed in 4% PFA overnight at 4 °C, embedded in paraffin, sectioned into 4 mm slices, dewaxed, dehydrated, stained with hematoxylin and eosin, and observed under a light microscope.

For Oil Red, larvae were fixed in 4% PFA overnight at 4 °C, washed with PBS, incubated with increasing concentrations of 1,2-propylene glycol, stained with oil red O solution overnight at 4 °C, rinsed, and washed with PBS.

For Nile Red, samples were fixed in 4% PFA overnight at 4 °C, washed with PBS, stained with 0.5 µg/ml Nile red for 10 min, washed, and stained with a nuclear dye at room temperature. The comprehensive procedure for H&E, Oil Red, and Nile Red staining of zebrafish is available in the previously published protocol [16, 17].

### Immunochemical and immunofluorescent staining

The comprehensive staining procedure is available in the earlier documented protocol [18, 19].

### Cell line and cultivation conditions

LO2, AML12 cells and RAW264.7 cells were cultured in DMEM containing 1% penicillin and 10% fetal bovine serum (FBS, Gibco, USA) at 37 °C, 5% CO_2_.

### Western blot analysis

Proteins were extracted from cells using ice-cold RIPA lysis buffer (Beyotime Biotechnology, China) with phosphatase inhibitors (Sigma, USA) and protease inhibitor cocktail (Sigma, USA). The samples were centrifuged for 15 min at low temperature and the supernatant was collected (12,000 rpm, 4 °C) after standing for 10 min in a 4 °C refrigerator. Quantification was performed using a BCA kit (Beyotime Biotechnology, Shanghai, China). Then we incubated the primary antibody overnight on a shaker at 4 °C, washed the strips the next day with TBST buffer, and incubated the secondary antibody for 2 h on a shaker at 4 °C, followed by chemiluminescence detection.

### Gene overexpression and knockdown in macrophages

For the purpose of siRNA transfection, 6-well plates were used to seed RAW264.7 cells. 250 µL of Opti-Medium containing 6 µL of Lipofectamine was mixed with 250 µL of Opti-Medium containing 5 µL of *Socs1*-siRNA. Next, 500 µL of the mix and 1500 µL of DMEM containing 10% FBS were added to each well and incubated for 4–6 h. Finally, medium was replaced with fresh growth medium.

To transfect the *Socs1* gene using lentivirus, RAW264.7 cells were placed in 96-well plates. According to the cell MOI and virus titer, the corresponding amount of virus was added to the 1.5 ep tube, and then added to the 96-well plates, cultured at 37 °C for 12–16 h, the medium was replaced. About 72 h after infection, the infection efficiency was observed. Uninfected cells were selected by adding puromycin.

### RNA extraction and qRT-PCR analysis

Total RNA was extracted using TRIzol reagent as per the manufacturer’s instructions. qRT-PCR was conducted on the ABI Prism 7500 Real-Time PCR System with the Prime Script RT Reagent Kit and SYBR^®^ Premix Ex Taq™ II, following manufacturer guidelines. PCR primers are listed in Tables 1 and 2. Data were normalized to GAPDH or β-actin levels, and mRNA expressions were calculated relative to GAPDH or β-actin using the 2 − ΔΔCt method.

**Table 1:** The PCR primers derived from mice

	Forward (5′ → 3′)	Reverse (5′ → 3′)
Il1a	CAGCCTTATTTCGGGAGTCTATTC	TATCCCTTTGTTAACCCATCTGTA
Il6	CAAAGCCAGAGTCCTTCAGAG	AGCATTGGAAATTGGGGTAG
Tnfα	TCTTCTCATTCCTGCTTGTGG	ATGAGAGGGAGGCCATTTG
Fasn	TGGTGGTGTGGACATGGTCACAGA	CCGAAGCTGGGGGTCCATTGTGTG
Apoa1	GCTTGGCACGTATGGCAGCA	TCTCCAGGTTATCCCAGAAG
Srebp1	AAGATGTACCCGTCCGTGTC	TGAAGGCAGGCTCGAGTAAC
Gapdh	TGGCAAAGTGGAGATTGTTG	CATTATCGGCCTTGACTGTG

**Table 2:** The PCR primers derived from zebrafish

	Forward (5′ → 3′)	Reverse (5′ → 3′)
Il1a	TTAACGAGGATCTGCATAAAGC	CATGTTCTCGTTGACCTTCTG
Il6	CCTCAAACCTTCAGACCGCT	GAACAGGATCGAGTGGACCG
Tnfα	GCTTATGAGCCATGCAGTGA	TGCCCAGTCTGTCTCCTTCT
fasn	GAGAAAGCTTGCCAAACAGG	GAGGGTCTTGCAGGAGACAG
srebp1	CATCCACATGGCTCTGAGTG	CTCATCCACAAAGAAGCGGT
apoa1a	GCACTAAGCTGACCGAGCGT	GGAGGTCCTGGGTGTGTGGA
SOCS1	ATGGTAGCACACAACCAGGT	AAATCTGGAAGGGGAAGGAGC
gapdh	TCGGTCGCATTGGC	CCGCCTTCTGCCTTA
β-actin	ATGGATGAGGAAATCGCTGCC	CTCCCTGATGTCTGGGTCGTC

### Statistical analysis

GraphPad Prism version 9.0 software is used to analyze all descriptive statistics, and numerical results are shown as mean ± standard deviation (mean ± SD). A t-test is suitable for comparing two samples, whereas one-way ANOVA is suitable for testing three or more samples. Statistical significance was determined by P values less than 0.05. ns., not significant. *p < 0.05, **p < 0.01, ***p < *0.001.* Each experimental result was repeated at least three times.

## Results

### The embryotoxic effects of BA concentrations in zebrafish

**Fig. 1:**
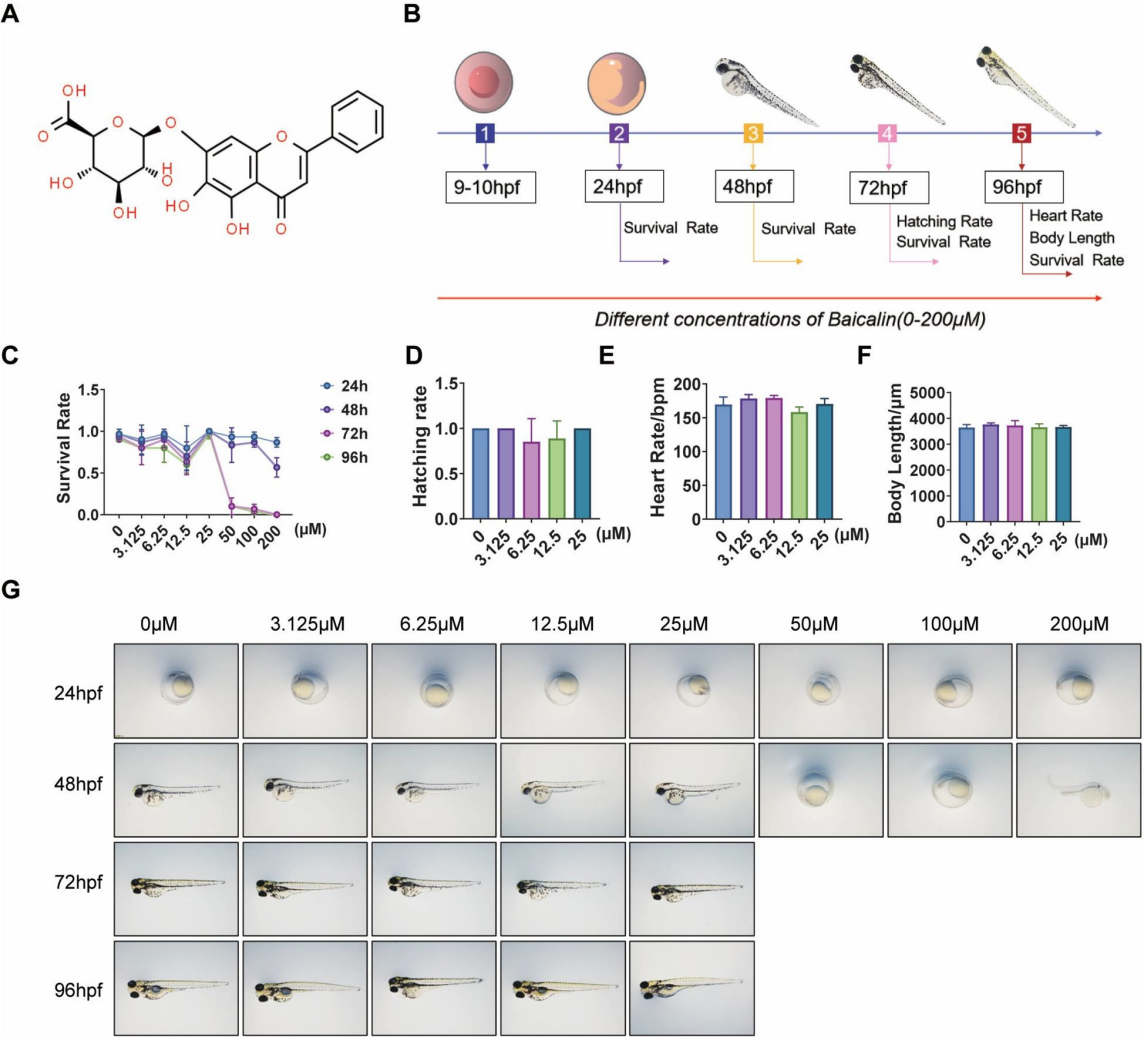
The toxicology of BA in zebrafish larvae.** A** Chemical structure of BA quoted from Pubchem. **B** Flowchart of treating zebrafish larvae with different concentrations of BA. **C** Effects of different concentrations of BA on the survival rate of zebrafish larvae. **D** Hatching rate of zebrafish larvae exposed to different concentrations of BA. **E** Heart rate of zebrafish larvae exposed to different concentrations of BA. **F** Body length of zebrafish larvae exposed to different concentrations of BA. **G** The developmental malformations in zebrafish larvae exposed to the indicated concentrations of BA for 96 h. The data are displayed as the means ± SD, n = 10

BA is a glycosyloxyflavone, specifically the 7-O-glucuronide derivative of baicalein. It is a key component of the Chinese herbal remedy *Scutellaria Baicalensis*, with a molecular weight of 446.4, as depicted in Fig. 1A. The study involved observing the survival rate, hatching rate, body length, heart rate, and morphological changes in zebrafish larvae treated with BA over a 96-h period. The experimental setup is depicted in Fig. 1B. The survival rate of zebrafish larvae at 72 and 96 hpf significantly dropped when exposed to BA concentrations of 50–200 μM (Fig. 1C). Between 48 and 72 hpf, nearly all embryos exposed to 0–25 μM BA successfully hatched into zebrafish larvae (Fig. 1D), suggesting that this concentration of BA does not adversely affect zebrafish hatching during development. There was no statistical difference in body length and heart rate tests of zebrafish larvae among the treatment groups from 0 to 25 µM (Fig. 1E and F). Zebrafish larvae exposed to 50–200 μM BA exhibited morphological changes such as pericardial edema, yolk retention, and a lack of swim bladder. Based on the above toxicity study results, we found that BA was safe at 0–25 µM. We chose 6.25 μM, 12.5 μM and 25 μM BA to treat ALD in zebrafish larvae induced by ethanol.

### BA mitigates alcoholic liver damage by decreasing lipid buildup in zebrafish larvae

**Fig. 2:**
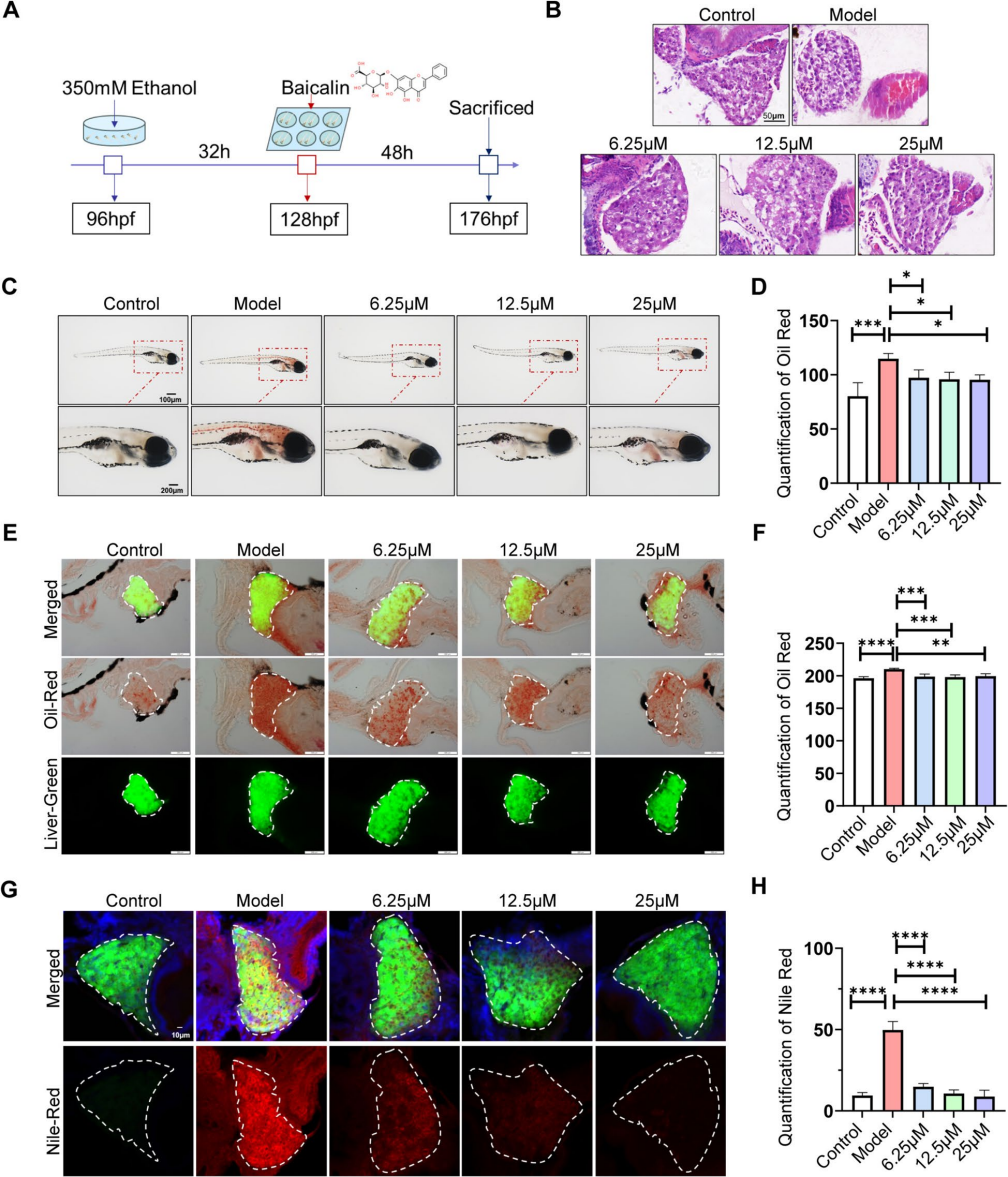
BA attenuated hepatic steatosis induced by alcohol in zebrafish larvae. **A** Process diagram for modeling ALD and BA drug treatment in zebrafish larvae. **B** H&E staining of the liver in zebrafish larva (scale bar, 50 μm). **C** Lipid droplets in the whole-mount zebrafish liver were stained with oil red O after BA treatment (scale bar, 100 μm, 200 μm). **D** Quantitative analysis of oil red O staining in zebrafish larvae (scale bar, 200 μm). **E** and **F** Oil Red O staining of frozen slices of zebrafish larvae and quantification. **G** and **H** Nile red staining of zebrafish larvae and quantification (scale bar, 10 μm). Data are expressed as the mean ± SD, n = 10. (*p < 0.05, **p < 0.01, ***p < 0.001, ****p < 0.0001)

Studies conducted previously have demonstrated that 4-day post-fertilization zebrafish larvae exposed to 350 mM ethanol for 32 h exhibit significant liver enlargement, vacuolization in liver tissue, and behavioral issues, along with induced oxidative stress. To create an alcoholic fatty liver model, we exposed 96 hpf zebrafish larvae to 350 mM ethanol for 32 h. Subsequently, the zebrafish model was treated with BA for 48 h (Fig. 2A). A significant aspect of alcoholic liver damage is lipid peroxidation. To determine if lipids had built up in the liver, hepatic tissues were examined using H&E, Oil red, and Nile red staining. The histopathological findings showed that BA treatment following ethanol exposure improved hepatic steatosis and repaired liver tissue structures in zebrafish larvae (Fig. 2B). In line with expectations, BA treatment significantly reduced the area and number of lipid droplets in the liver parenchyma of zebrafish larvae, as detected by Oil Red staining, compared to the model group (Fig. 2C and D). Furthermore, Oil Red staining of frozen liver sections produced similar outcomes (Fig. 2E and F). Nile Red staining of frozen liver sections indicates a protective effect in reducing lipid accumulation in the liver of larvae following ethanol exposure (Fig. 2G and H). Our study revealed that 6.25, 12.5, and 25 µM BA have therapeutic effects on alcohol-induced liver injury, with 25 µM being the most effective concentration for combating steatosis in zebrafish models.

### BA attenuates alcoholic induced liver injury by repressing hepatic steatosis in vitro

**Fig. 3:**
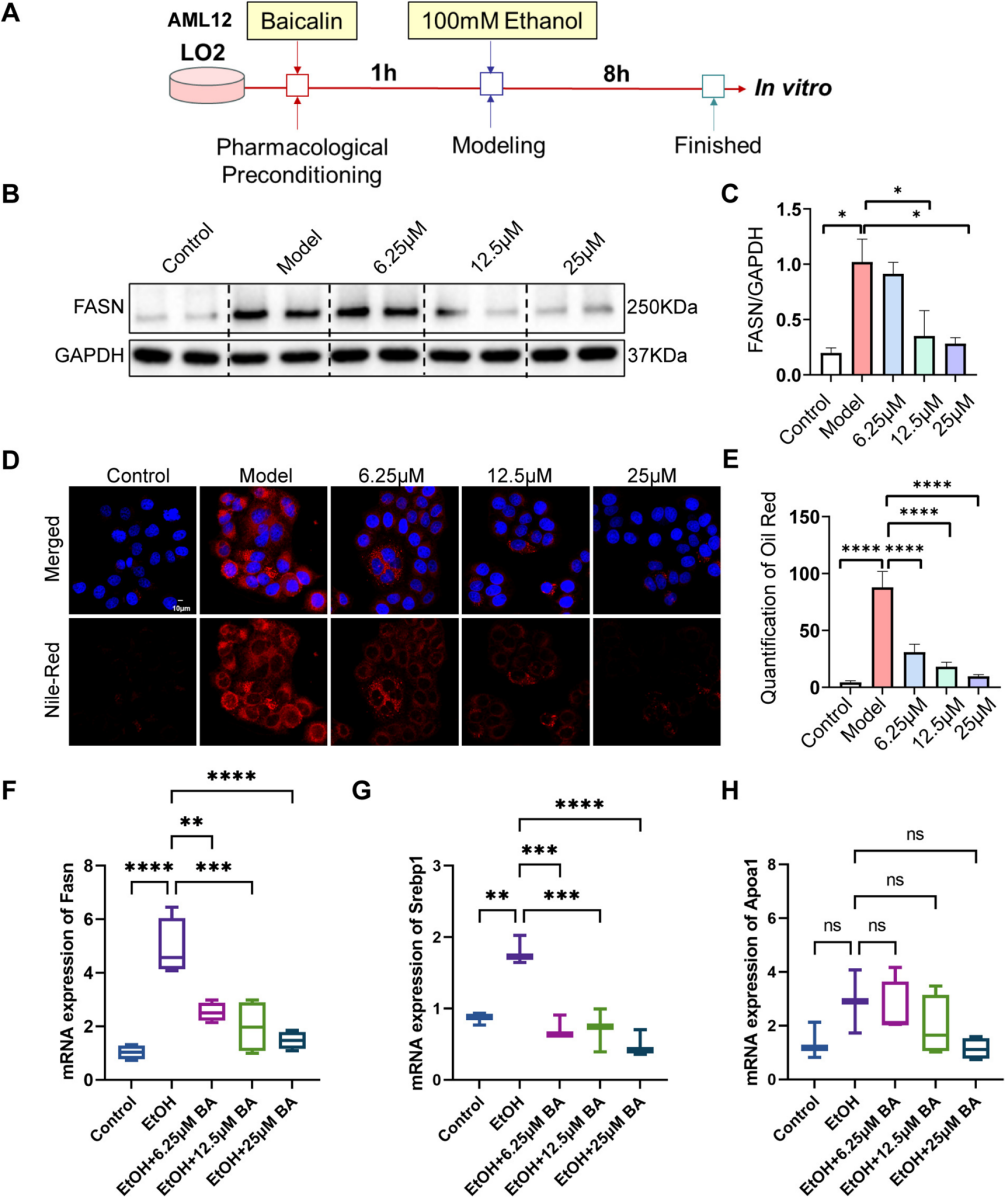
BA reduced lipid accumulation in LO2 and AML12 cells induced by alcohol. **A** Process diagram for modeling ALD and BA drug treatment in LO2 and AML12. **B** Western-blot detection of FASN protein expression in control, model group, and 6.25 μM BA, 12.5 μM BA, 25 μM BA drug group with 100 mM ethanol treatment. **C** Quantitative analysis of FASN. **D** Oil red O staining of LO2 slides in control, model group, and 6.25 μM BA, 12.5 μM BA, 25 μM BA drug group with 100 mM ethanol treatment, scale bar, 10 μm. **E** Quantitative analysis of oil red O staining in LO2. **F–H** qPCR detection of Fasn, Srebp1 and Apoa1 gene expression in AML12 cells from different groups. Data are expressed as the mean ± SD, n = 3–6 per group. (*p < 0.05, **p < 0.01, ***p < 0.001, ****p < 0.0001)

Conversely, we simulated alcoholic liver cell damage in vitro by treating LO2 cells with 100 mM ethanol for 8 h, based on earlier research. Prior to the 8-h ethanol exposure, LO2 was pretreated with BA for 1 h (Fig. 3A). In line with the zebrafish experiments mentioned earlier, the in vitro ALD model was employed to examine the effectiveness of BA in combating liver steatosis. Fatty acid synthase (FASN) plays a crucial role in controlling the de novo fatty acid synthesis pathway. Western Blot analysis revealed a significant increase in FASN expression in the model group, whereas it decreased in the 12.5 and 25 µM BA groups (Fig. 3B and C). Meanwhile, we also found that BA could alleviate the upregulation of *Fasn* gene in zebrafish induced by alcohol (Fig.S1). Nile Red staining revealed that treating LO2 with 100 mM ethanol for 8 h significantly increased intracellular lipid accumulation. However, BA at concentrations of 6.25 µM, 12.5 µM, and 25 µM reduced this lipid accumulation (Fig. 3D and E). Furthermore, we re-established an in vitro model of alcoholic fatty liver in AML-12 cell line. The results of qRT-PCR showed that compared with the alcohol-treated group, after treatment with BA, the expression of *Fasn* was significantly inhibited, thus reducing lipid synthesis. In addition, the detection results of *Srebp1* indicated that the expression of *Srebp1* was significantly increased in the model group, while after intervention with BA, the expression of *Srebp1* decreased, thereby reducing fatty acid synthesis. The therapeutic effect of BA was significantly concentration-dependent, and the concentration of 25 µM had the best effect. In addition, we also detected the expression of *Apoa1*, and the results showed that there was no statistical difference among the control group, the model group and the drug BA-treated group (Fig. 3F–H). The findings suggested that BA counteracted alcoholic liver damage by reducing lipid build up in the liver. Additionally, BA was most effective at concentrations between 6.25 and 25 µM, with 25 µM being the optimal concentration.

### BA reduced alcoholic liver injury by reducing macrophages accumulation and inducing macrophage polarization towards M2

**Fig. 4:**
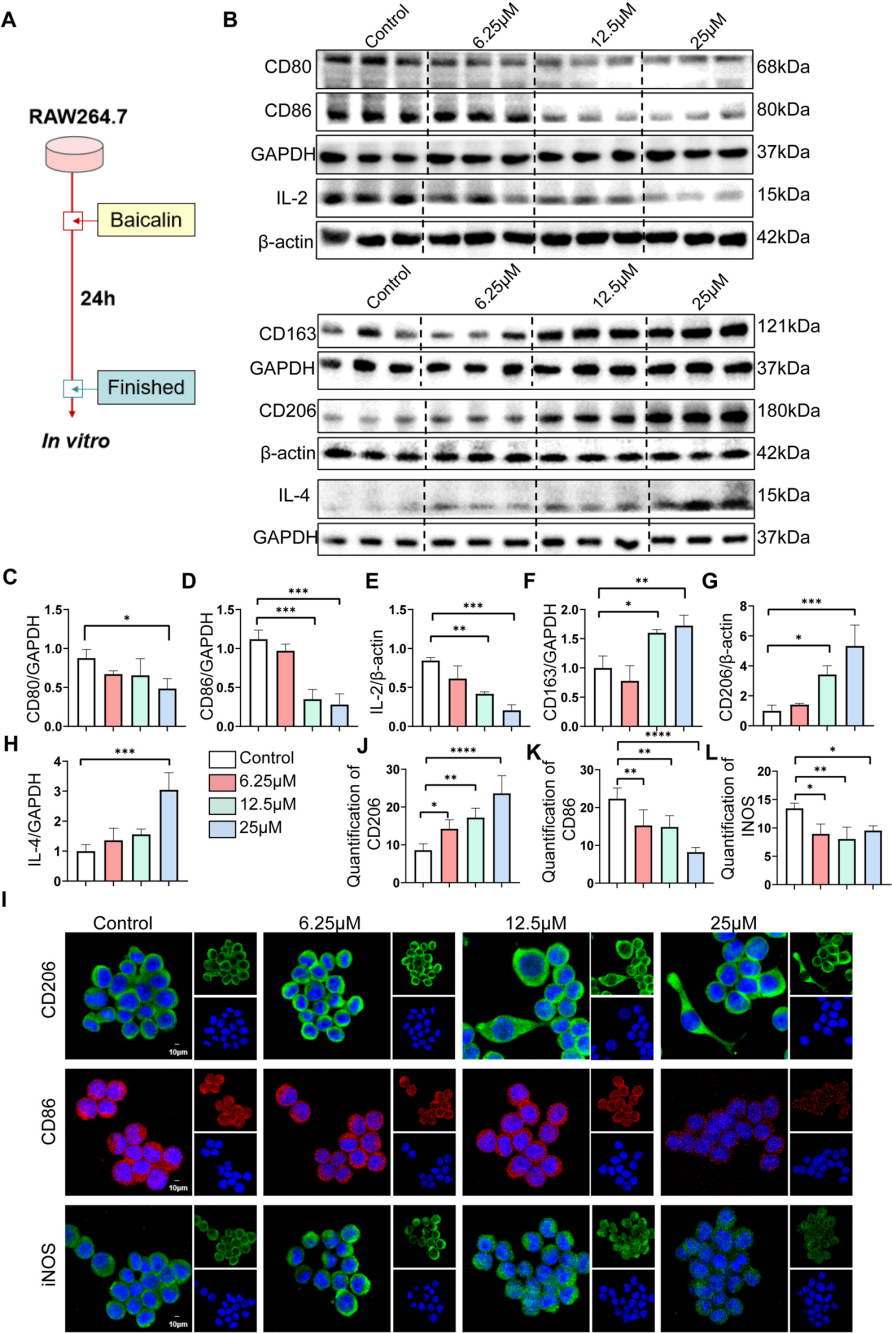
BA induced phenotypic polarization of RAW264.7 cells to M2-type cells in vitro. **A** RAW264.7 cells were exposed to control, 6.25 μM BA, 12.5 μM BA, 25 μM BA for stimulation respectively. **B** Protein immunoblotting of CD206, CD86, IL-2, CD163, CD206, and IL-4 expression levels in the control group and 6.25μM BA, 12.5μM BA, 25μM BA drug group. **C–H** Image J software analysis of CD80, CD86, IL-2, CD163, CD206, IL-4 expression levels vs. GAPDH or β-actin. **I** Immunofluorescence staining of CD206, CD86 and iNOS expression in the control group and 6.25 μM BA, 12.5 μM BA, 25 μM BA drug group. **J–L** Quantification of the mean fluorescence intensity of CD206, CD86 and iNOS using image J software. All data are shown as mean ± SD. n = 3–6 per group. Scale bar, 10 μm. (*p < 0.05, **p < 0.01, ***p < 0.001, ****p < 0.0001)

Next, we examined how BA affected macrophage phenotype. Initially, we used *Tg(mpeg1:mCherry)* zebrafish to eatablish ALD model and added BA treatment. We found that compared to the control group, the expression of *Socs1* gene in zebrafish from the model group was significantly downregulated, while BA could upregulate the expression of *Socs1* in ALD zebrafish (Fig.S2 A). Interestingly, we discovered through fluorescence microscopy that a large number of macrophages clustered near the liver in ALD zebrafish, while the accumulation of macrophages near the liver was significantly reduced after drug treatment (Fig.S2B). In addition, we also extracted RNA from zebrafish for qPCR detection. Interestingly, we found that in the model group, the expression of *IL-6*, *IL-1*, and *Tnf-α* genes in zebrafish were significantly upregulated, while the expression levels of these inflammatory genes were significantly downregulated after BA drug treatment (Fig.S2 C-E). The above results collectively indicate that BA may alleviate inflammatory response by regulating macrophage accumulation, thereby reducing lipid accumulation. RAW264.7 cells were exposed to BA for 24 h to examine its impact on their polarization (Fig. 4A). The findings showed that BA reduced the levels of CD80, CD86, and IL-2 (Fig. 4B–E), while it elevated the levels of CD163, CD206, and IL-4 in RAW264.7 cells (Fig. 4F–H). Immunofluorescence results showed that the expression of CD206 was increased after BA treatment while the expression of CD86 and iNOS were decreased (Fig. 4I–L). In addition, BA also inhibited inflammation related genes (such as* IL-6*, *IL-1* and *Tnf-α*) in macrophages (Fig.S3). According to the data, The phenotypic markers for M2 macrophages included CD163, CD206, and IL-4, while those for M1 macrophages were CD80, CD86, and IL-2. The findings collectively imply that BA contributed to the formation of alternative plasticity in macrophages and facilitated their shift towards the M2 phenotype to lessen alcoholic liver injury.

### BA transformed LPS or alcohol-induced M1 macrophages into M2-type phenotype in a laboratory setting

**Fig. 5:**
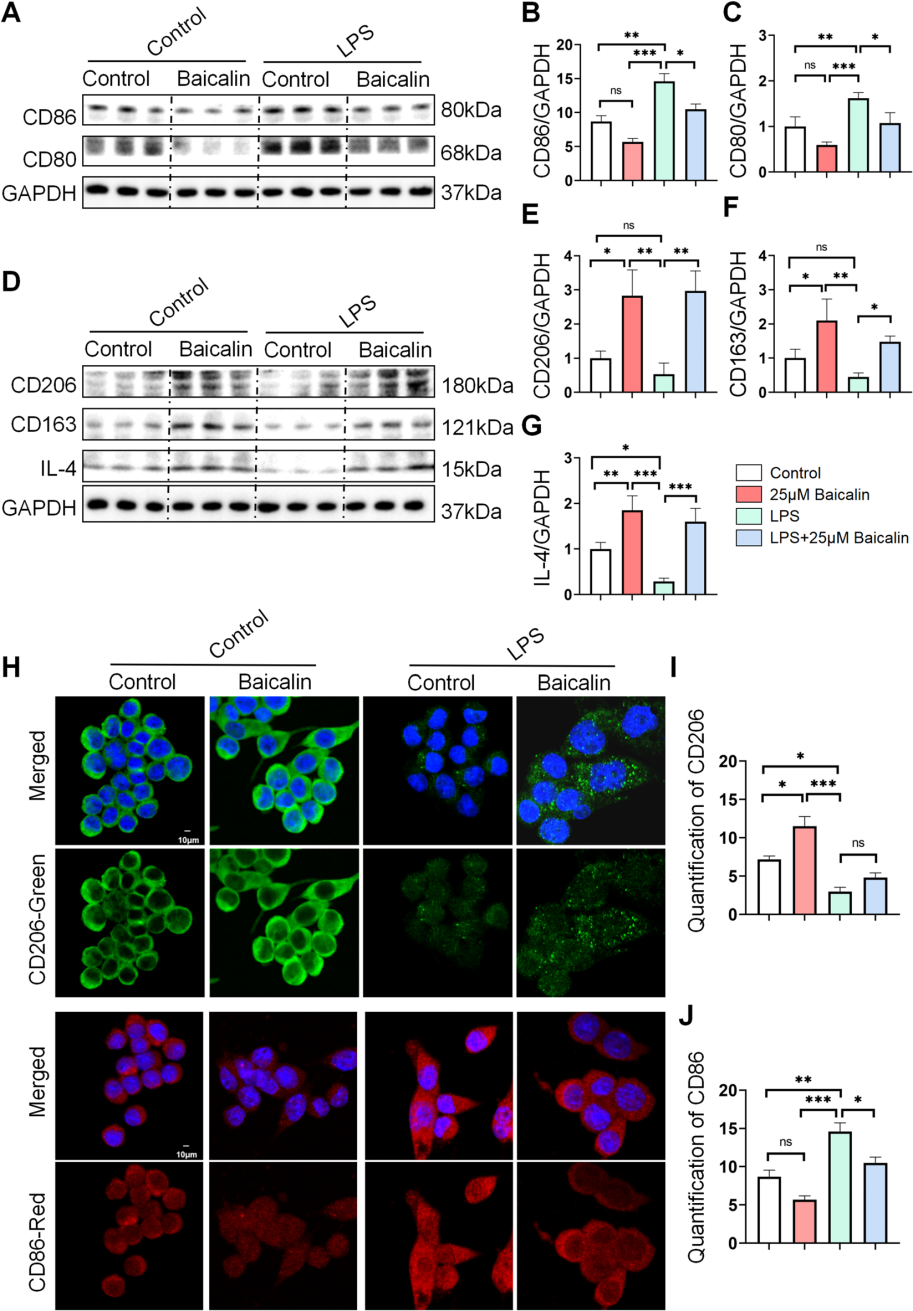
BA could promote the reprogramming of LPS-induced M1-type macrophages to an anti-inflammatory M2-type cell phenotype in vitro. RAW264.7 cells were stimulated by exposure to control, 25 μM BA, LPS and their conditioned medium, respectively. **A** Western blot detection of CD86 and CD80 expression levels in the control group, 25 μM BA group, LPS group, and LPS + 25 μM BA group. **B, C** Quantification of expression levels of CD86 and CD80 vs. GAPDH using image J software. **D** Western blotting detection of CD206, CD163 and IL-4 expression levels in the control group, 25 μM BA group, LPS group, and LPS + 25 μM BA group. **E**–**G** Quantification of expression levels of CD206, CD163 and IL-4 vs. GAPDH using image J software. **H** Immunofluorescence staining of CD206 and CD86 expression in the control group, 25 μM BA group, LPS group, and LPS + 25 μM BA group. **I, J** Quantify CD206 and CD86 expression in immunofluorescence staining using image J software. All data are shown as mean ± SD. n = 3–6 per group. Scale bar, 10 μm. (*p < 0.05, **p < 0.01, ***p < 0.001, ****p < 0.0001)

Next, we looked into the effects of M1 stimuli (LPS) and 25 µM BA on the phenotype of macrophages. Initially, M0 cells exposed to 100 µg/mL LPS were polarized into the M1 phenotype, after which 25 µM BA was introduced for 24 h to examine its impact on M1 cell phenotype repolarization. The findings showed that LPS stimulation of M0 cells led to an increase in CD80 and CD86 expression, but the group treated with LPS + BA was able to significantly lower this increase (Fig. 5A–C). In contrast, CD206, CD163, and IL-4 expression rose in the BA and LPS + BA-treated groups, with no differences noted in the untreated and LPS groups (Fig. 5D–G). Accordingly, the immunofluorescence analysis showed a significant upregulation of CD206 in the BA group, with a decrease observed in the LPS and LPS + BA-treated groups. CD86 expression was significantly increased in the LPS group (Fig. 5H–J). Meanwhile, we constructed an alcohol-induced macrophages model in vitro, and the results were consistent with above LPS-induced macrophages. After alcohol stimulation, the expression of CD206 in macrophages decreased, while the expression of CD80 increased, indicating that alcohol can facilitate macrophages polarization towards M1 in ALD. However, BA can inhibit macrophages polarization towards M1 type (Fig.S4 A-B). In addition, we also examined the expression of SOCS1 and found that the expression of SOCS1 was downregulated after alcohol induction, while BA could upregulate the expression of SOCS1 (Fig.S4 C-D). Similarly, after alcohol induction, the expression level of inflammatory factors in macrophages significantly increased in the alcohol group, while the expression level of inflammatory factors significantly decreased in the BA treatment (Fig.S4 E–G). Generally, these data revealed that BA repolarized LPS or alcohol-induced M1 macrophages to M2-type phenotype.

### BA influences the shift of M1 macrophages to the M2 phenotype by controlling *Socs1* expression in vitro

**Fig. 6:**
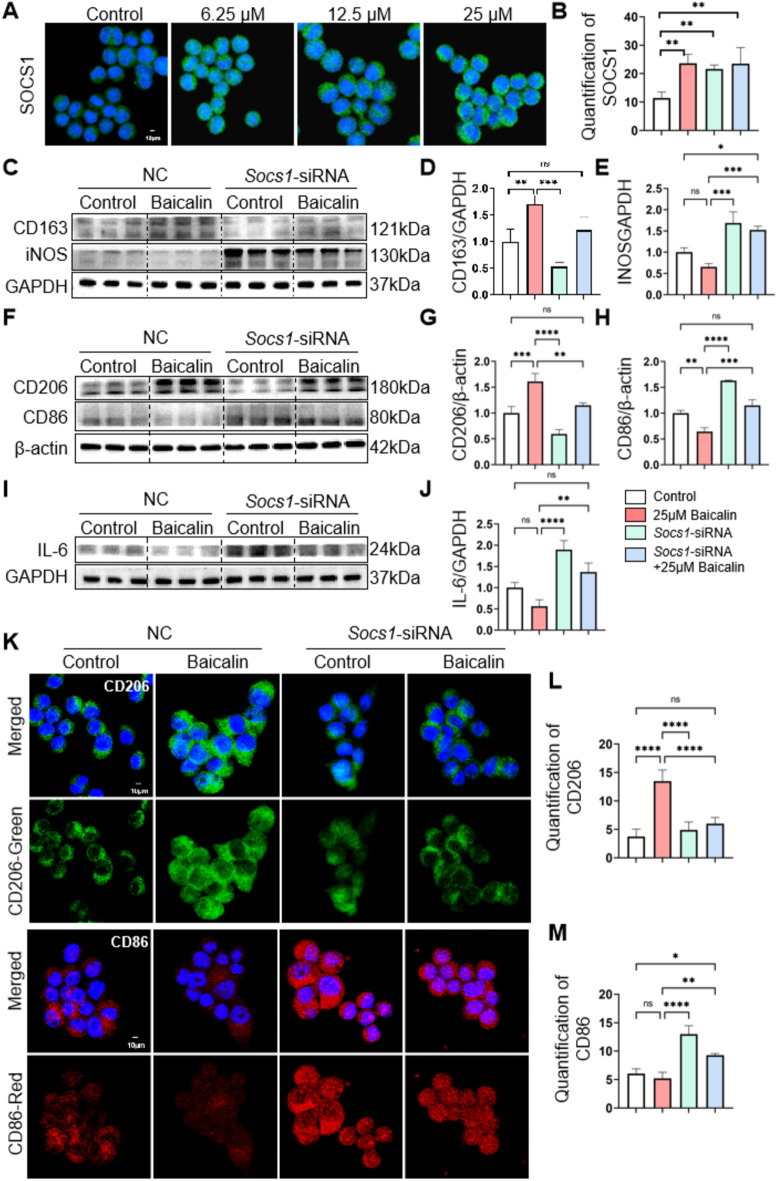
Silencing *Socs1* enhanced the polarization of RAW264.7 cells to the M1-type, whereas BA induces the repolarization of M1-type macrophages to the M2-type. The *Socs1*-siRNA RAW264.6 cells were constructed to block SOCS1 expression. Untreated RAW264.7 cells and *Socs1*-siRNA RAW264.7 cells were exposed to control, 25 μM BA and their conditioned medium for stimulation, respectively. **A**, **B** The effect of SOCS1 protein overexpression was analyzed by immunofluorescence staining and the quantification of SOCS1 in the control group and 6.25 μM BA, 12.5 μM BA, 25 μM BA drug group. **C**–**E** Western blot analysis of protein expression levels of CD163 and iNOS in the control group, 25 μM BA group, *Socs1* siRNA group, and *Socs1*-siRNA + 25 μM BAgroup and quantification of expression levels of CD163 and iNOS vs. GAPDH using image J software. **F**–**H** Western blot analysis of protein expression levels of CD206 and CD86 in the control group, 25 μM BA group, *Socs1* siRNA group, and *Socs1*-siRNA + 25 μM BAgroup and quantification of expression levels of CD206 and CD86 vs. β-actin using image J software. **I**, **J** The expression of IL-6 in the control group, 25 μM BA group, *Socs1* siRNA group, and *Socs*1-siRNA + 25 μM BAgroup was analyzed by western blotting and quantified with image J software. **K** Immunofluorescence staining of CD206 and CD86 expression in the control group, 25 μM BA group, Socs1 siRNA group, and *Socs1*-siRNA + 25 μM BA group. **L**, **M** Quantify CD206 and CD86 expression in immunofluorescence staining using image J software. All data are shown as mean ± SD. n = 3–6 per group. Scale bar, 10 μm. (*p < 0.05, **p < 0.01, ***p < 0.001, ****p < 0.0001)

In our in vivo experiments, SOCS1 expression was higher following BA drug treatment compared to the control group. To validate the influence of SOCS1 on RAW264.7 cells polarization, we effectively developed a RAW264.7 cells line with *Socs1* knocked down. Upon knocking down *Socs1*, we found that the levels of M2-related factors like CD206 and CD163 were notably reduced in the BA + *Socs1*-siRNA group compared to the BA group (Fig. 6C, D, F and G). The BA + *Socs1*-siRNA group exhibited significantly elevated levels of M1-related factors like iNOS, CD86, and IL-6 compared to the BA group (Fig. 6E and H–J). Likewise, the immunofluorescence staining results indicated that the CD206 level was significantly reduced in the BA + *Socs1*-siRNA group compared to the BA group (Fig. 6K–L), while the level of the CD86 was considerably higher in the BA + *Socs1*-siRNA group (Fig. 6M). The results indicate that knocking down *Socs1* inhibited RAW264.7 cells from polarizing into the M2-type phenotype, showing that SOCS1 is essential for BA-induced macrophage polarization towards the M2 type.

### *Socs1* overexpression encouraged RAW264.7 cells to polarize into the M2 phenotype in vitro

**Fig. 7:**
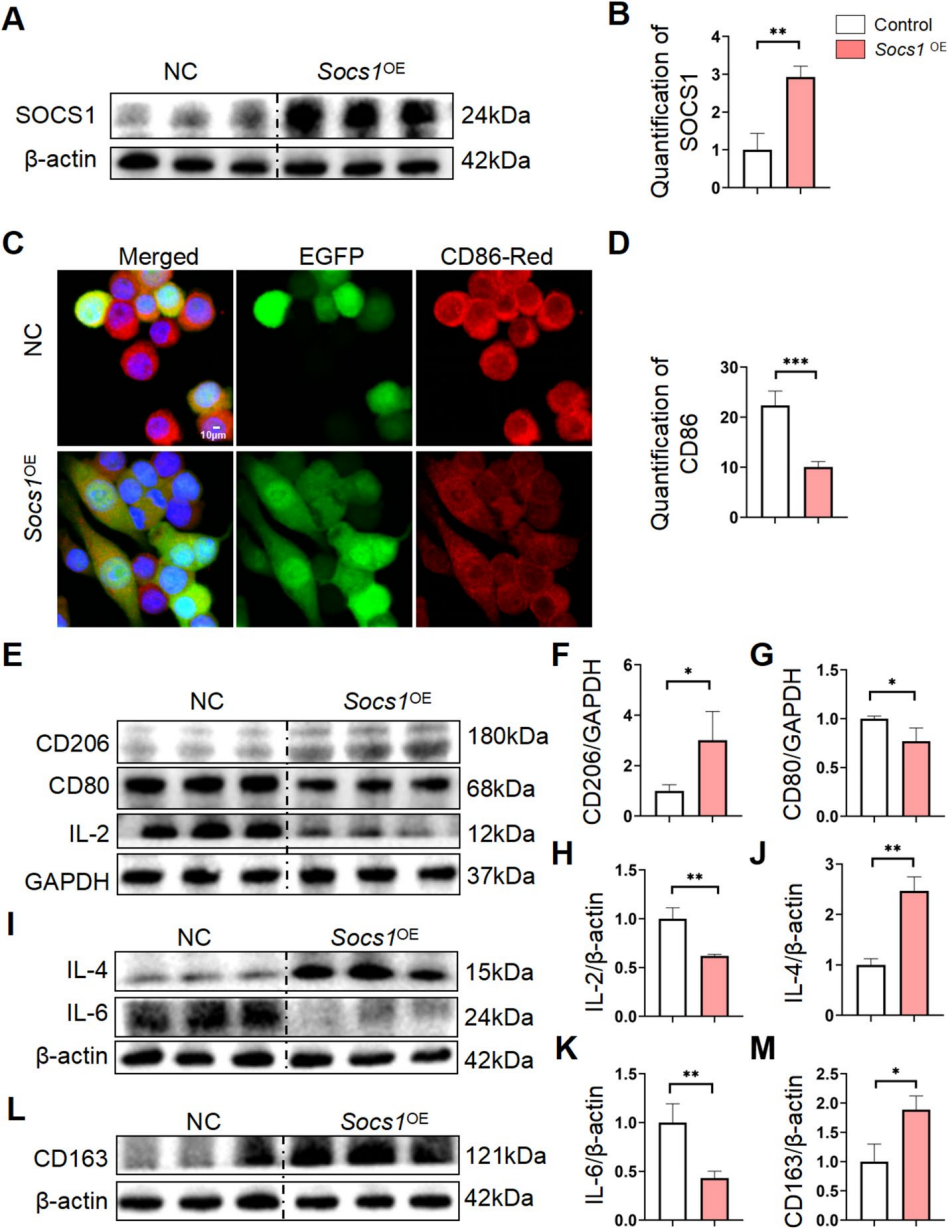
Socs1 overexpression promoted M2-type macrophage polarization while inhibiting M1-type macrophage polarization in RAW264.7 cells. **A**, **B** The protein overexpression effect of SOCS1 was analyzed by western blotting, and their quantification using image J software. **C**, **D** Protein expression levels of CD86 were analyzed by immunofluorescence staining and their quantification. **E**–**H** Western blot analysis of the expression of CD206, CD80 and IL-2 and the results of these proteins vs. GAPDH. **I**–**K** The expression of IL-4 and IL-6 were analyzed by western blotting and their quantification. **L**, **M** The protein expression levels of CD163 were analyzed by western blotting and quantified with image J software. All data are shown as mean ± SD. n = 3–6 per group. Scale bar, 10 μm. (*p < 0.05, **p < 0.01, ***p < 0.001, ****p < 0.0001)

According to earlier in vivo and in vitro findings, SOCS1 is essential in how BA influences M2 macrophage repolarization to mitigate alcoholic liver damage. Initially, as illustrated in Fig. 7A, B, we effectively developed RAW264.7 cells with overexpressed *Socs1*. Furthermore, immunofluorescence results indicated that CD86 expression was reduced in the *Socs1* overexpression group compared to the control group (Fig. 7C and D). Moreover, the western-blot assay showed an increase in M2-related factors (like CD206, IL-4, CD163) in the *Socs1* overexpression group compared to the untreated cells (Fig. 7E, F, I, J and L, M). Compared to the untreated group, the *Socs1* overexpression group showed reduced expression of M1-related factors such as CD80, IL-2, and IL-6 (Fig. 7G, H and K). From the data presented, we found that *Socs1* overexpression in RAW264.7 cells led to polarization towards the M2-type cell phenotype. In summary, our findings indicate that BA influences SOCS1 expression to encourage macrophage polarization in vitro.

## Discussion

**Fig. 8:**
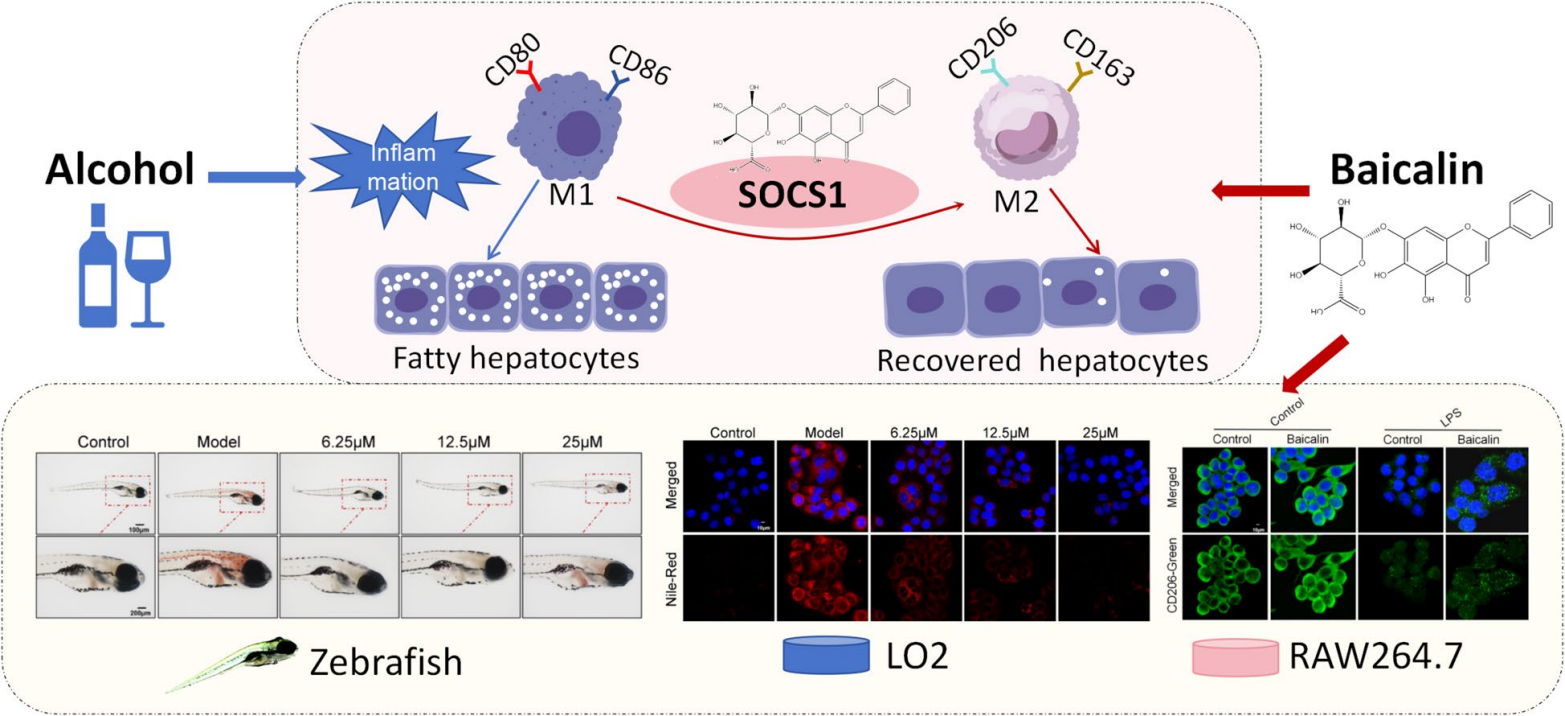
.

Despite numerous studies highlighting the liver-protective effects of BA, the underlying molecular mechanisms remain uncertain. The study’s data confirmed that BA inhibits the progression of ALD and started to uncover the mechanisms through which BA alleviates ALD. Due to the crucial role of macrophages in regulating inflammation, they have been considered to be involved in the pathogenesis of ALD. Meanwhile, this study provides new evidence that BA upregulates the expression of SOCS1, thereby promoting macrophage polarization towards M2 type, reducing inflammation, and finally slowing down the progression of ALD (Fig. 8).

It is commonly acknowledged that BA possesses a broad spectrum of pharmacological characteristics, such as antioxidation [20]. In depth research on molecular mechanisms suggests that long-term alcohol consumption impairs the ability of liver cells to respond to protective signals and makes these signals sensitive to inflammatory signals [21]. Moreover, more research has demonstrated the anti-inflammatory and antioxidant properties of BA in liver disease [22], indicating that it is a promising plant-based drug that can be used to prevent ALD. Consistently, our results confirmed the hepatoprotective effect of BA by alleviating ALD symptoms. In addition, our study also indicated that BA enhanced the expression of SOCS1 to inhibit the progression of ALD.

SOCS1 proteins attach to specific cytokine receptors, janus kinases, and signaling molecules to manage signaling pathways, thereby controlling immune and inflammatory reactions [23]. Within macrophages, it is capable of inhibiting the JAK-STAT signaling pathway of several cytokine receptors (including IL-6 receptor, IFN-γ receptor), thereby modulating the intensity and duration of immune and inflammatory responses [24]. Nonetheless, macrophages exhibit considerable diversity and polarization traits, which are deemed essential for the onset and progression of ALD inflammation. Macrophage polarization is primarily categorized into M1 and M2 types. M1 macrophages produce pro-inflammatory cytokines like iNOS and IL-6 and express proteins such as CD86 and CD80, whereas M2 macrophages express anti-inflammatory markers like CD206, CD163 and IL-4 [25, 26]. Research has shown that long-term alcohol consumption may disrupt the polarization balance of M1/M2 macrophages, significantly affecting the pathogenesis of ALD [27–29]. The study demonstrated a notable rise in M1 macrophages and a marked reduction in M2 in ALD mice, but SOCS1 reversed these outcomes, suggesting that SOCS1’s anti-inflammatory effect on ALD is due to its regulation of M1/M2.

In addition, the use of zebrafish for efficacy evaluation and mechanism research is also a distinctive feature of this study [30]. Zebrafish have a short life cycle and high reproductive rate. Their genetic material is highly similar to that of humans, and their bodies are transparent, as an emerging model for studying adipose tissue metabolism [31]. Besides, zebrafish have a transparent body during the first 5 days of development, which can be used for drug toxicity evaluation [32]. In previous studies, we have also successfully constructed a zebrafish model of ALD [17]. In this study, we first determined the safe administration concentration of BA and successfully constructed a zebrafish ALD model. We also found that BA can effectively inhibit fat accumulation in the liver of zebrafish. Furthermore, we constructed a liver cell ALD model in vitro experiments to evaluate the drug efficacy of BA. Finally, through in vitro RAW264.7, it was found that BA could regulate macrophage polarization through SOCS1, thus elucidating the mechanism by which BA alleviates ALD. Importantly, our study also investigated the potential relationship between macrophage polarization and hepatocytes by establishing a co-culture system using the Transwell platform, combining RAW264.7 macrophage cells and AML-12 hepatocyte cells (Fig.S5). The experimental results demonstrated that treatment with alcohol or LPS significantly increases lipid accumulation in hepatocytes. However, when treated with BA, the polarization of inflammatory macrophages was effectively reduced, leading to a significant decrease in lipid accumulation within hepatocytes, as the expression of *Fasn* and *Srebp1* genes were significantly decreased. These results indicates BA significantly promotes the migration and differentiation of macrophages into M2-type macrophages by increasing the expression of SOCS1. This, in turn, remarkably strengthens the repair function of macrophages and ultimately effectively alleviates the lipid deposition within hepatocytes. However, the limitation of this study is the inability to directly construct *Socs1* gene modified zebrafish mutants in vivo, and the inability to directly use primary cells in vitro experiments. In the next step of research, we will further explore the pharmacological mechanism of BA.

## Conclusion

Our study’s findings support the idea that liver macrophages are crucial in the progression of alcoholic liver damage. Our experiments indicate that BA facilitates the transformation of M1 macrophages into anti-inflammatory M2 macrophages by enhancing SOCS1 expression, thereby reducing inflammation damage. These findings provide robust pre-clinical evidence that supports BA as a potential alternative medication.

## Supplementary Information

Additional file 1

## Data Availability

The data associated with this study can be obtained from the corresponding author upon reasonable request.

## Abbreviations

ALD: Alcohol-associated liver diseases
AH: Alcohol-associated hepatitis
BA: Baicalin
SOCS1: Suppressor of cytokine signaling 1
MCD: Methionine and choline-deficient
NAFLD: Nonalcoholic fatty liver disease
FBS: Fetal bovine serum
PBS: Phosphate buffered saline
siRNA: Small interfering RNA
SD: Standard deviation
