# Supplementary material for: Baicalin mitigates alcoholic-associated liver disease via SOCS1-driven reprogramming of macrophages
Source: Chin Med. 2025 May 13;20:63. doi: 10.1186/s13020-025-01110-4 (PMC12070616; doi:10.1186/s13020-025-01110-4)
Supplement: Supplementary file 1 — Additional file 1 [file 13020_2025_1110_MOESM1_ESM.pdf]

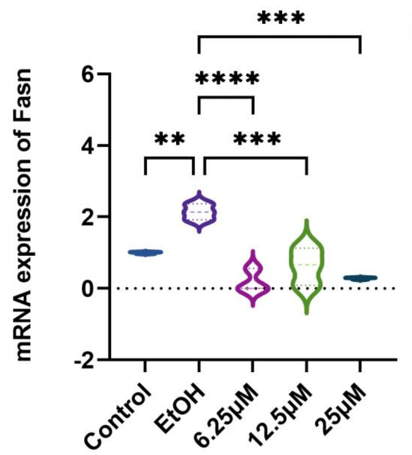

Fig. S1. BA regulates *Fasn* gene expression in ALD zebrafish.

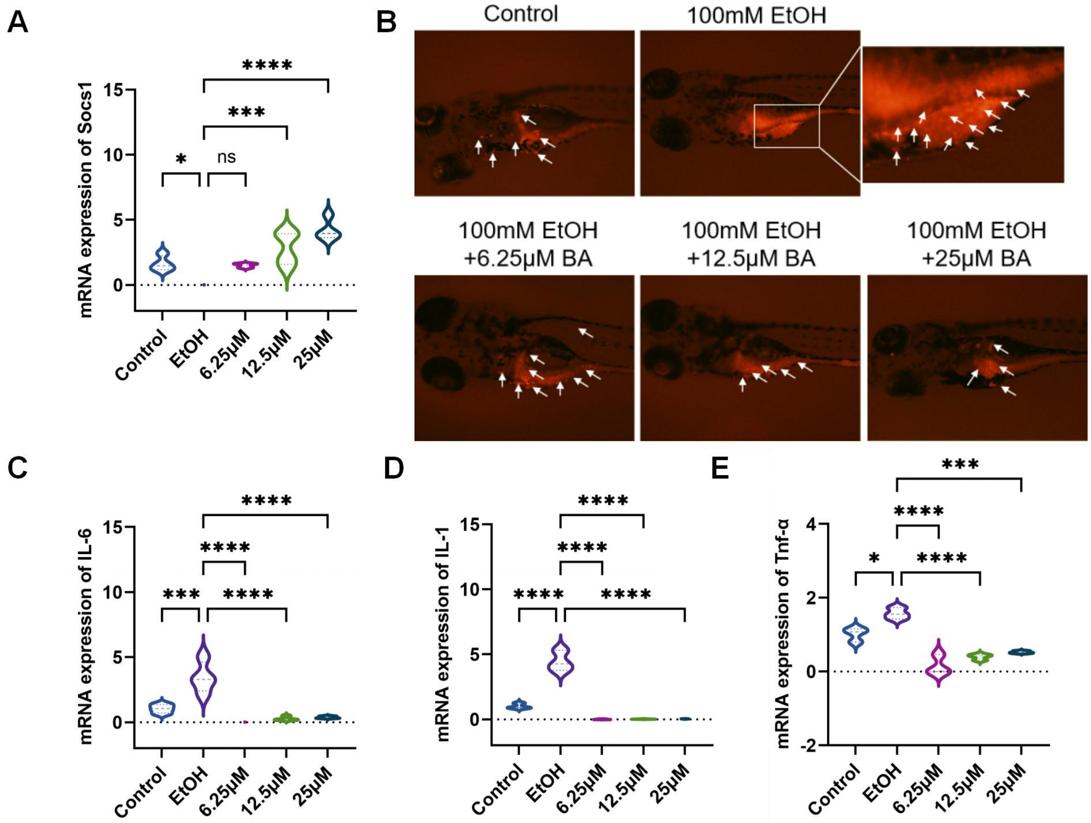

Fig.S2.BA reduces inflammation in zebrafish.

A. qPCR detection of *Socs1* gene expression in control, EtOH, EtOH+6.25μM BA, EtOH+12.5μM BA, and EtOH+25μM BA group of zebrafish. B.Fluorescence detection of macrophages in zebrafish of each group; C-E. qPCR detection of *IL-6, IL-1 and Tnf-α* gene expression in zebrafish from different groups.

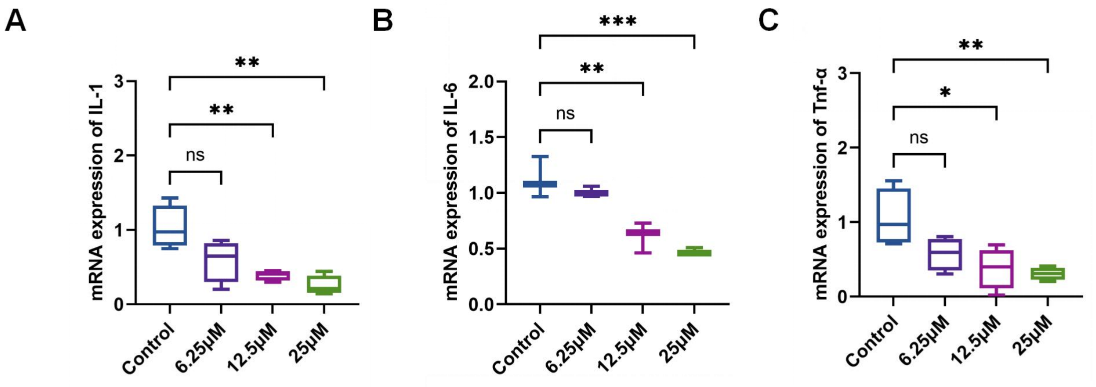

Fig. S3 .BA regulates *IL-6, IL-1 and Tnf-a* gene expression in macrophages.

A-C. qPCR detection of *IL-6, IL-1 and Tnf-α* gene expression in RAW264.7 cells from different groups.

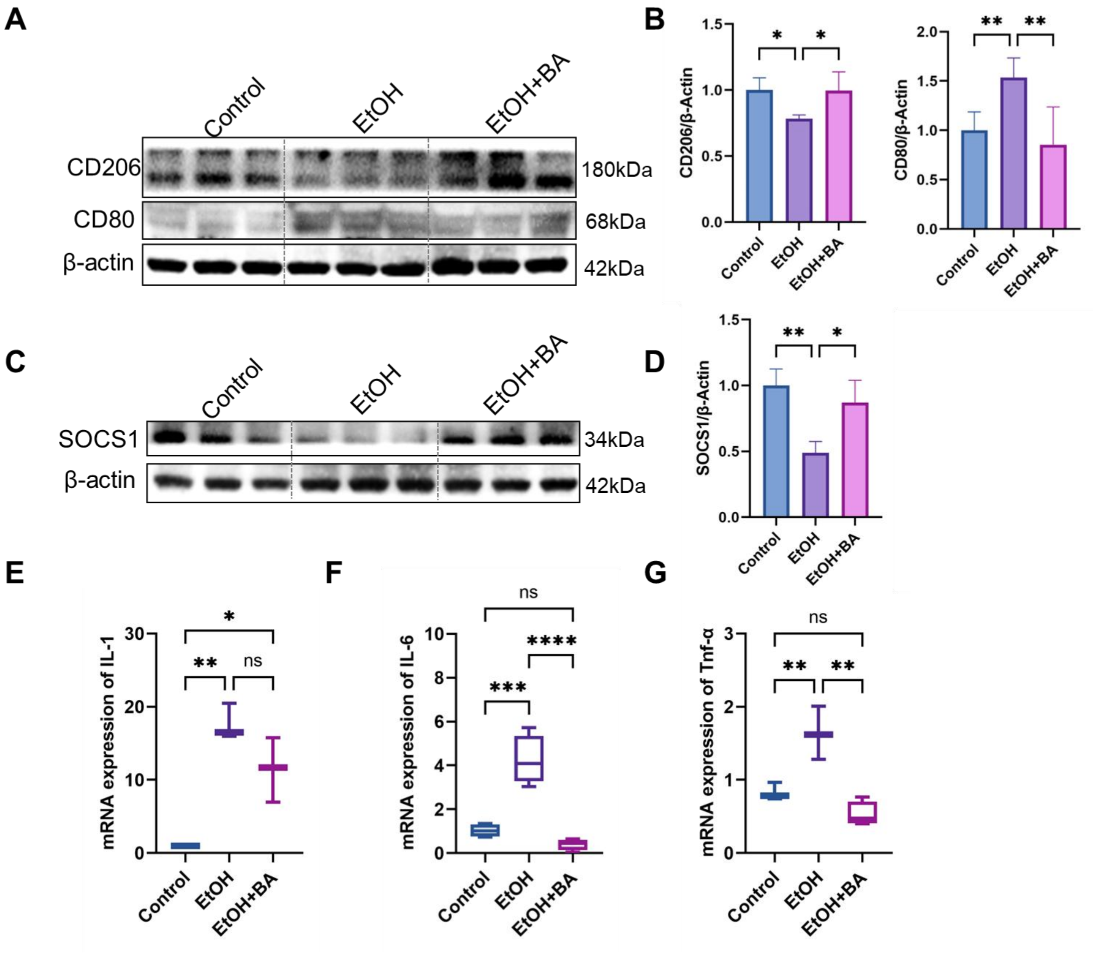

Fig.S4. BA promotes the reprogramming of EtOH-induced M1-type macrophages to an anti-inflammatory M2-type cell phenotype *in vitro*.

A. Western blot detection of CD206 and CD80 expression levels in the control group,

EtOH (100mM) group, and EtOH+25μM BA group. B. Quantification of expression levels of CD206 and CD80 vs.β-actin using image J software. C. Western blotting detection of SOCS1 expression levels in the the control group, EtOH group, and EtOH+25μM BA group. D. Quantification of expression levels of SOCS1 vs..β-actin using image J software. E-G.qPCR detection of IL-6, IL-1 and Tnf-α gene expression in RAW264.7 cells from different groups.

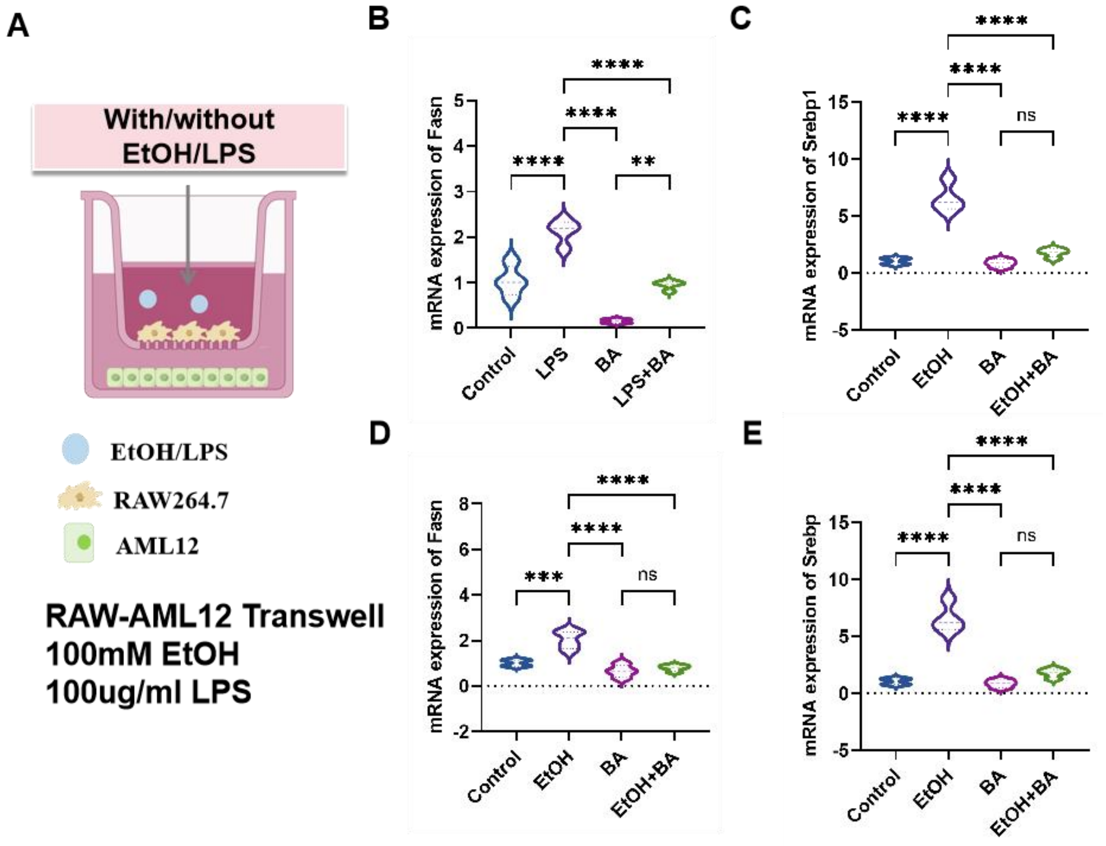

Fig.S5. BA mitigates ALD by decreasing lipid accumulation influencing macrophages polarization .

A.RAW-AML12 co culture processing flowchart. B-C.qPCR detection of *Fasn* and *Srebp1* gene expression in the supernatant of AML12 co cultured with LPS-induced RAW with or without BA. D-E.qPCR detection of *Fasn* and *Srebp1* gene expression in the supernatant of AML12 co cultured with EtOH-induced RAW with or without BA.
